# Mycotoxin patulin contamination in various fruits and estimating its dietary impact on the consumers: From orchard to table

**DOI:** 10.1016/j.heliyon.2024.e30252

**Published:** 2024-04-28

**Authors:** Shahzad Z. Iqbal, Muhammad Waseem, Ahmad Faizal Abdull Razis, Ijaz A. Bhatti, Amin Mousavi Khaneghah, Osama A. Mohammed, Srimathi Priya Lakshminarayanan, Munawar Iqbal

**Affiliations:** a Food Safety and Toxicology Lab., Department of Applied Chemistry, Government College University Faisalabad, 38000, Pakistan; b Department of Food Science, Faculty of Food Science and Technology, Universiti Putra Malaysia, 43400 UPM Serdang, Selangor, Malaysia; c Department of Chemistry, University of Agriculture, Faisalabad, 38040, Pakistan; d Faculty of Biotechnologies (BioTech), ITMO University, 191002, 9 Lomonosova Street, Saint Petersburg, Russia; e Department of Pharmacology, College of Medicine, University of Bisha, Bisha, 61922, Saudi Arabia; f Department of Natural Resource Management, Horticultural College and Research Institute (HC & RI), Tamil Nadu Agricultural University (TNAU), Periyakulam, 625604, India; g Department of Chemistry, Division of Science and Technology, University of Education, Lahore, Pakistan; h Laboratory of Food Safety and Food Integrity, Institute of Tropical Agriculture and Food Security, Universiti Putra Malaysia, 43400 UPM Serdang, Selangor, Malaysia

**Keywords:** Mycotoxins, Patulin, Fruits, Dietary intake, Processing steps, Risk assessment

## Abstract

The present research examined patulin's presence across the whole supply chain of selected fruits. A comprehensive analysis was conducted on 442 samples of fruits (oranges, apples, apricots, lemons, and guava) to determine the presence of patulin contamination. This analysis used Liquid Chromatography (HPLC) with a UV detector. The findings indicate that 17, 23, and 28 % of selected fruit samples tested positive for patulin levels in farm, transportation, and market samples. However, the sample collected during the transportation step showed that 56 % (percentage of positive samples) of fruits have patulin levels greater than 50 μg/kg, and 41 % (percentage of positive samples) have greater levels than 50 μg/kg in market samples. The findings of the one-way analysis of variance indicated that no statistically significant variation existed between the amounts of patulin across the various stages of the food supply chain system (p > 0.05). Nevertheless, the analysis of the correlation study, namely Kendall's tau_b and Spearman's rho, denote a robust association between the levels of patulin and the food supply system. The apple samples exhibited the most significant average dietary intake of patulin, with an average value of 0.11 μg/kg bw/day. The maximum mean hazard quotient (HQ) of 0.28 was also recorded. The prevalence and incidence of patulin in specific fruits were found to be relatively high, and it was observed that market samples had elevated levels of patulin in the selected fruits.

## 1 Introduction

Mycotoxins are classified as natural harmful secondary metabolites primarily synthesized by certain filamentous fungi under suitable humidity and temperature settings [[1], [2], [3]]. Patulin is classified as a dangerous secondary mycotoxin [4]. Its synthesis is attributed to around 60 different harmful fungi, including *Penicillium expansum* (*P. expansum*), *Penicillium patulum* (*Penicillium griseofulvum*), and *Penicillium crustosum* [5]. According to previous research, it has been indicated that *Penicillium expansum* is the predominant fungus responsible for the production of patulin [6]. According to Ostry et al. [7], the International Agency for Research on Cancer has classed the toxicity of patulin as a group 3 (not carcinogen to humans). Several research studies have shown evidence of hazardous toxic health effects such as genotoxic, teratogenic, and carcinogenic qualities to living organisms [[8], [9], [10], [11]].

Previous research has reported that the consumption of this substance has been associated with the promotion of gastrointestinal and neurological complications [12]. This phenomenon's impact on living beings manifests in various ways, such as the impairment of DNA integrity through oxidative damage, the development of micronuclei, and the occurrence of chromosome abnormalities [10]. According to Saleh and Goktepe [13], elevated patulin levels are associated with adverse effects in several demographic groups, including individuals of different genders, races, and population groupings.

To avoid its exposure and health hazards worldwide, different organizations have implemented strict regulations on patulin in food and other commodities [14]. WHO (World Health Organization) [15] has established a 50 μg/kg maximum limit in apple juice, ingredients, and beverages. The EU (European Union) has implemented a 50 μg/kg maximum limit for patulin in various juices. Apple products used for baby foods are 10 μg/kg, and solid apple products have a limit of 25 μg/kg [16].

Numerous studies have shown evidence that mycotoxin contamination constitutes a substantial concern to the food supply chain, particularly concerning industrial food and feed. This contamination jeopardizes food safety and animal health and has implications for international trade [[12], [17], [18]]. Mycotoxins in food can occur at several points across the food supply chain, including cultivation, transit, storage, and processing [19]. Hence, the presence of these substances in food is a crucial matter, with notable implications for both health and the country's economy. Pakistan is internationally acknowledged for producing high-quality fruits cultivated under tropical or subtropical environmental conditions that remain favorable year-round. According to the government report from 2019 [20], the cultivation of fruits spans an expansive region of 800,000 ha, resulting in a substantial production of 7.05 million tons.

The prevalence of patulin in fruits and juices has been extensively testified in prior research. Hence, the main emphasis of the current project was to investigate the prevalence of patulin in the supply chain network, specifically in samples obtained from several stages spanning from the farm to the market, with a particular focus on chosen fruits. Furthermore, the research also focuses on ascertaining and comparing the concentrations of patulin in different fruits to the rules set by the European Union. Additionally, the study seeks to quantify the dietary intake of patulin among local consumers.

## 2 Methodology

### 2.1 Sampling

**Fig. 1:**
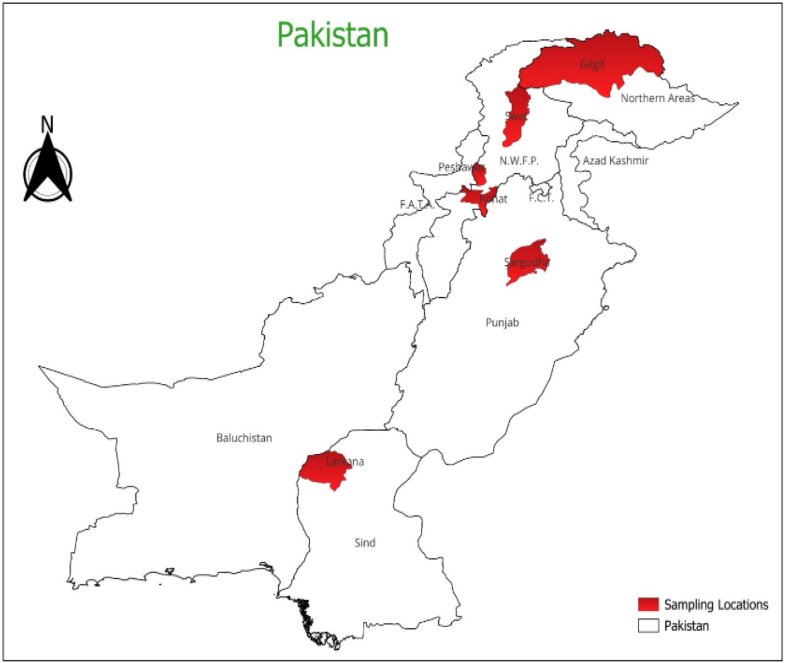
Sampling site of selected fruits from different areas of Pakistan.

Four hundred forty-two fruit samples, including oranges, apples, apricots, lemons, and guava, were collected between June 2022 and December 2022. These samples were obtained from three different sources: 140 samples were directly collected from farms, 141 samples were acquired during the transportation of these fruits from farms to the market, and 161 samples were collected from marketplaces in chosen cities. The journey duration from cities such as Gilgit and Naran typically spans a period of one to two days, during which goods are transported in vehicles that lack proper temperature and moisture control. The sampling regions are depicted in Fig. 1. The transportation samples were obtained from 61 batches of fruits, verified, and validated by the respective farm owners. The sample size was consistently maintained at a minimum of 1 kg, and each sample was securely packaged in polyethylene plastic bags. Subsequently, the samples were placed in a freezer and kept at 4 °C.

### 2.2 Regents and chemicals

The patulin standard (100 μg/mL, Merck, Beijing, China) in acetonitrile was already available in the lab, and reagents such as ethyl acetate, sodium acetate, acetic acid, and sodium carbonate were acquired from Merck and Sigma-Aldrich (France). Fisher Scientific (France) has provided high purity solvents such as methanol and acetonitrile. The deionized water (Merck Millipore) was used during the analysis. Furthermore, the quality of other chemicals was ensured to be analytical grade.

### 2.3 Extraction of patulin

The procedure of extracting patulin from fruits was followed by a method [21] with some changes. The whole fruit was homogenized with peels, and then the sample (50 g) was mixed with 100 mL water and 50 mL ethyl acetate and homogenized for 15 min with the help of a vortex mixer. Then, the solution was centrifuged at 4500 rpm for 10 min, and the organic layer was again transferred to a centrifuge tube. The aqueous layer was again extracted twice with ethyl acetate (20 mL). Then, all layers (organic) were mixed, and 2 mL sodium carbonate (1.5 %) was added and shaken vigorously. After shaking, 5 mL of ethyl acetate was added to the tube and shaken vigorously for 5 min. The pH 4 of the solution was maintained with the help of glacial acetic acid, and then it was dried with the help of a nitrogen stream to dryness at a temperature of 60 °C. Then, the residue was dissolved in 5 mL of 5 % acetonitrile solution. Moreover, the prepared solution was purified through a 0.22 mm syringe filter (Millipore). Again, the filtered solution was evaporated to dryness at 60 °C, 500 μl solvent of pure methanol was added, and 20 μl sample was subjected to LC analysis.

### 2.4 HPLC conditions

The system was a Shimadzu LC-10 A series, Kyoto, Japan, with a UV detector. The column was of Discovery HS, Merck, Bellefonte, USA (C18, 3 × 15, 4.6 mm, 5 mm). The mobile phase's isocratic mode consisted of (90: 10) % acetonitrile and water, operating with a 1.5 mL/min flow rate. The detector's wavelength was set at 276 nm.

### 2.5 Dietary intake estimation

The dietary intake (DI) of patulin was estimated in selected fruits following the previous method [22]. The participants of 480 were chosen to fill out the fruit frequency questionnaire according to their weekly consumption. The participant's mean weight was 66 ± 1.5 kg. The inclusion and exclusion criteria for selecting participants were based on their knowledge about food consumption and some primary knowledge of food safety. The dietary intake was estimated based on the relation shown in Eq. (1).

(1) $$\text{Dietary}\;\text{intake}\;\text{of}\;\text{patulin}\;\left(\mu g/\text{kg}\;\text{bw}/\text{day}\right)=\frac{Consumption\;of\;fruits\;\left(\frac{g}{day}\right)x\;patulin\;level\;\left(\mu \frac{g}{kg}\right)}{weight\;\left(kg\right)}$$

### 2.6 Risk assessment

The risk assessment of patulin due to the ingestion of fruits was estimated following the method described by Torovic et al. [23]. The relation is shown in Eq. (2).

(2) $$\text{HQ}=\frac{DI\;\frac{g}{kg}/day}{PMTDI\;\left(\mu \frac{\frac{G}{KG}}{DAY}\right)}$$

Where HQ is the Hazard Quotient; DI = dietary intake; PMTDI is the provisional maximum tolerance daily intake of patulin as 0.4 μg/kg BW/day [24].

### 2.7 Quality control parameters

The quality control parameters such as linearity, repeatability, reproducibility, recovery analysis, limit of detection (LOD), and limit of quantification (LOQ) were determined. The recovery analysis was analyzed by spiking 6 concentrations in blank samples of apples (25, 50, 200, 400, 600, and 800 μg/kg). The linearity was checked by constructing a calibration curve (7 points) for PAT from 5 to 1200 μg/L. The repeatability was assessed by inserting the standard (15 μg/L) before the start of the analysis, during the middle of the analysis, and after the analysis. The reproducibility was determined by comparing standard and reference material results with the collaborating lab. The concentration of patulin in samples were determined using calibration curve. The LOD was calculated as a 3:1 signal-to-noise ratio, and LOQ was determined as a 10:1 signal-to-noise ratio.

### 2.8 Statistical analysis

The data was given as mean ± SD, and the samples below LOD and greater than zero were replaced with $\frac{LOD}{\sqrt{2}}\text{.}$ The normality of the distribution of data was analyzed by plotting q-q graphs. One-way ANOVA was used to distinguish between fruit type, processing process, and patulin levels. LSD was used to investigate the significant difference between groups at p ≤ 0.05. Correlation analysis, especially Kendall's tau_b and Spearman's rho, were analyzed to distinguish the interaction of different factors. Box and whisker plots were constructed using SPSS (IBM, 29.5, USA).

## 3 Results

### 3.1 Quality control parameters

The results of the recovery analysis varied from 79.8 to 104. 5 %, with an average relative standard deviation of 9–16 %. The coefficient of determination (R^2^) has shown an excellent value of 0.9996, which ultimately reflects that the standard curve was a good fit and the system was linear. The LOD and LOQ were 0.16 and 0.49 μg/kg, respectively.

The LOD and LOQ documented in the current study were relatively high compared to earlier studies [25,26]. Pernica et al. [27] have reported the levels of LOD and LOQ of 4.9 and 6.6 μg/L of patulin. The values of LOD from 2.6 to 7.5 μg/kg and LOQ of 8.0–15.0 μg/kg were documented in dried fruits, juices, and jams samples [4]. The values of 3 to 1 μg/L of LOD and 10 to 1 μg/L of LOQ were determined in fruit juices and wine samples for patulin [28]. The levels of LOD and LOQ were quite comparable with the values of other studies.

### 3.2 Patulin assessment during postharvest processing steps

**Fig. 2:**
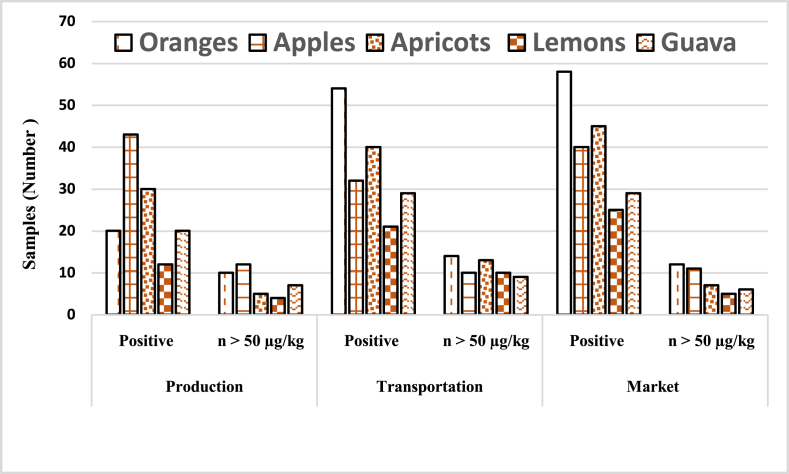
Graphical representation of patulin positive and sample greater than 50 μg/kg, in selected fruits during processing steps.

In the current study, 442 samples of fruits (oranges, apples, apricots, lemons, and guava) were analyzed during postharvest steps for patulin, and the results are represented in Table 1. The findings revealed that 75, 102, and 124 samples of fruits were observed to be contaminated with patulin in farm, transportation, and market samples, respectively. The highest mean of 75.24 ± 39.6 μg/kg was found in fruit samples analyzed in transportation samples. Fig. 2 represents the positive samples and samples greater than 50 μg/kg maximum limit in fruit samples in farm samples, transportation samples, and samples collected from the market. The results documented that a high frequency of samples were contaminated during transportation.

**Table 1:** Assessment of patulin (μg/kg) levels in selected fruits from the food supply chain system.

		a Farm Samples			Transportation Samples			Market Samples		
**Samples type**	**Total Samples**	Positive (%)^a^	Concentration (μg/kg)	Range (μg/kg)	Positive (%)	Concentration (μg/kg)	Range (μg/kg)	Positive (%)	Concentration (μg/kg)	Range (μg/kg)
Oranges	124	20	75.50 ± 6.50	LOD- 130.75	34	95.70 ± 9.30	LOD- 170.70	38	95.80 ± 5.50	LOD- 110.15
Apples	98	15	110.75 ± 4.30	LOD- 210.50	20	135.40 ± 2.80	LOD- 250.70	22	145.70 ± 8.20	LOD- 269.40
Apricots	80	10	45.60 ± 2.60	LOD- 90.45	15	55.50 ± 4.50	LOD- 106.50	20	50.64 ± 5.64	LOD- 94.50
Lemons	50	10	35.50 ± 3.50	LOD- 75.78	11	43.50 ± 4.50	LOD- 78.80	18	45.55 ± 5.50	LOD- 61.70
Guava	90	20	36.70 ± 4.50	LOD- 90.80	22	46.10 ± 5.50	LOD- 95.50	26	44.78 ± 6.50	LOD- 120.70
	442	75 (17 %)	60.8 ± 32.3		102 (23 %)	75.24 ± 39.6		124 (28 %)	77.10 ± 44.4	

^a^total number of samples percentage.

a Farm samples = field samples.

**Fig. 3:**
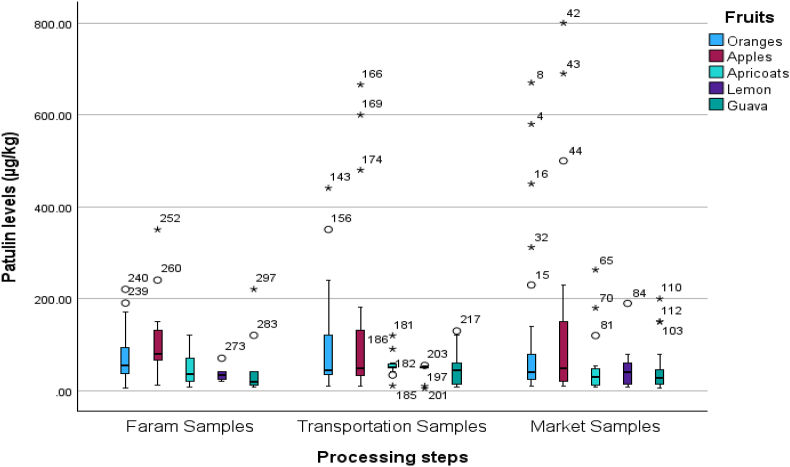
Box and whisker plot of patulin during processing steps, patulin levels.

**Fig. 4:**
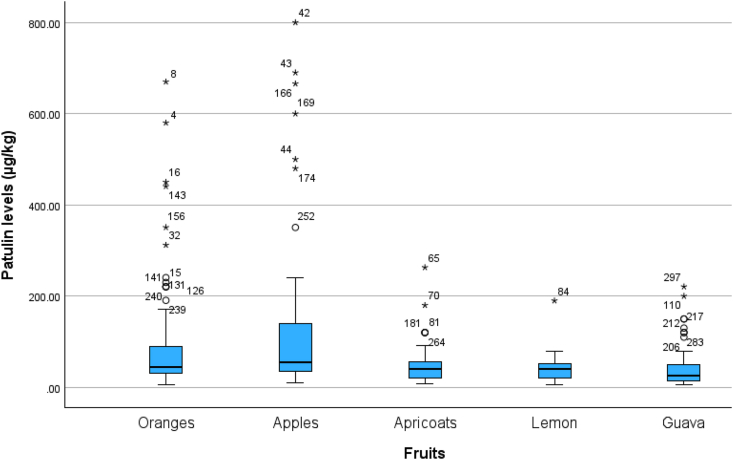
The selected fruit samples categories (*Represents the outlier, ° = Values that are more than 1.5 times the interquartile range away from the box are considered to be outliers and shown as circles).

The normal distribution of patulin levels from normal values was evaluated by plotting Q-Q plots as represented in Fig. 3, Fig. 4. Fig. 3 shows the deviation of the observed value to normal, represented by Fig. 4, that the data was aligned on the negative side and left side skewed distribution. That means the data is not normally distributed.

**Fig. 5:**
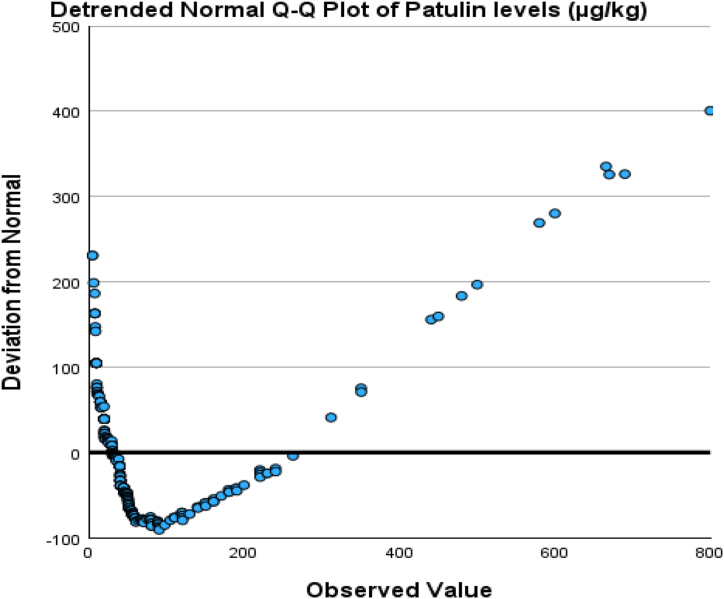
The q-q plot about normality distribution between normal value and original data.

**Fig. 6:**
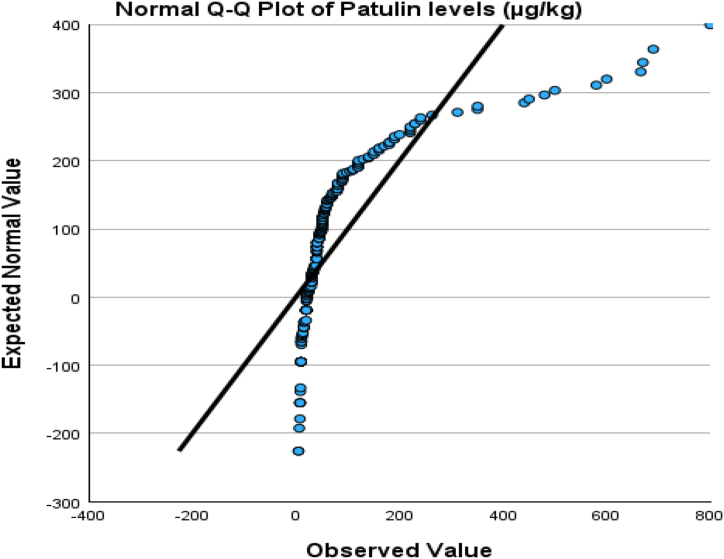
The q-q plot about normality distribution between the expected value and original data.

Hence, a one-way analysis of variance (ANOVA) was analyzed to examine the significant variation of patulin across different fruits and processing processes. The results of this analysis are given in Table 2a, Table 2b. The examination findings implied no significant relationship between the levels of patulin and the types of fruits (p > 0.05). However, a significant association was seen between the levels of patulin and the processing steps (p < 0.01). Hence, an analysis of variance (ANOVA) was conducted to assess the statistical significance of the various processing processes and their impact on the levels of patulin in fruits. The findings of this analysis can be found in Table 2(a), while the post-hoc LSD test results are presented in Table 2(b). The findings indicate that no statistically significant variations were seen in the amounts of patulin throughout different processing steps involved in its transportation from the farm to the market. Correlation analysis is a more rational approach in this scenario, as indicated by its inclusion in Table 3. Kendall's tau_b and Spearman's rho revealed a significant negative association between the kind of fruits and the number of processing stages involved, with correlation coefficients of −0.198 and −0.265, respectively. Furthermore, these tests also demonstrated a noteworthy association. The box and whisker plot against patulin levels and processing steps (Fig. 5) and between patulin levels and different types of fruits (Fig. 6) explained the range of data in the current study.

**Table 2a:** One-way analysis of variance between patulin levels and food supply chain system.

ANOVA					
Patulin levels (μg/kg)					
	Sum of Squares	df	Mean Square	F	Sig.
Between Groups	17142.585	2	8571.292	0.672	0.512
Within Groups	3802094.901	298	12758.708		
Total	3819237.486	300

**Table 2b:** LSD comparison between Patulin and processing steps involved during transportation of selected fruits.

Multiple Comparisons							
Dependent Variable: Patulin levels (μg/kg)							
	(I) Processing steps	(J) Processing steps	Mean Difference (I-J)	Std. Error	Sig.	95 % Confidence Interval	
						Lower Bound	Upper Bound
LSD	Faram Samples	Transportation Samples	−18.39529	17.18143	0.285	−52.2076	15.4170
		Market Samples	−16.47640	16.52298	0.319	−48.9929	16.0401
	Transportation Samples	Faram Samples	18.39529	17.18143	0.285	−15.4170	52.2076
		Market Samples	1.91890	15.09895	0.899	−27.7952	31.6330
	Market Samples	Faram Samples	16.47640	16.52298	0.319	−16.0401	48.9929
		Transportation Samples	−1.91890	15.09895	0.899	−31.6330	27.7952

**Table 3:** Correlation analysis of patulin levels and food supply chain system.

Correlations					
			Processing steps	Fruits	Patulin levels (μg/kg)
Kendall's tau_b	Processing steps	Correlation Coefficient	1.000	−0.025	−0.081
		Sig. (2-tailed)	.	0.612	0.071
		N	301	301	301
	Fruits	Correlation Coefficient	−0.025	1.000	−0.198^a^
		Sig. (2-tailed)	0.612	.	<0.001
		N	301	301	301
	Patulin levels (μg/kg)	Correlation Coefficient	−0.081	−0.198^a^	1.000
		Sig. (2-tailed)	0.071	<0.001	.
		N	301	301	301
Spearman's rho	Processing steps	Correlation Coefficient	1.000	−0.030	−0.106
		Sig. (2-tailed)	.	0.610	0.066
		N	301	301	301
	Fruits	Correlation Coefficient	−0.030	1.000	−0.265^a^
		Sig. (2-tailed)	0.610	.	<0.001
		N	301	301	301
	Patulin levels (μg/kg)	Correlation Coefficient	−0.106	−0.265^a^	1.000
		Sig. (2-tailed)	0.066	<0.001	.
		N	301	301	301

a Correlation is significant at the 0.01 level (2-tailed).

### 3.3 Dietary intake of patulin in fruits

Table 4 denotes the estimated dietary intake (μg/kg/day) and HQ levels in fruit samples from different processing steps. The findings revealed the highest mean dietary intake of 0.11 μg/kg/day in apple samples collected during the marketplace, with an HQ value of 0.28. The HQ levels varied from 0.026 to 0.11.

**Table 4:** Dietary intake (μg/kg bw/day) of patulin in selected fruits.

		Oranges	Apples	Apricots	Lemons	Guava
**Farm Samples**	Consumption (g/day)	27	50	25	40	50
	Patulin (μg/kg)	75.5	110.7	45.5	35.4	36.7
	Dietary Intake (μg/kg/day)	0.03	0.08	0.02	0.02	0.03
	HQ	0.08	0.21	0.04	0.05	0.07
**Transportation**	Consumption (g/day)	27	50	25	40	50
	Patulin (μg/kg)	95.6	135.6	55.5	43.5	46.1
	Dietary Intake (μg/kg/day)	0.04	0.102	0.021	0.026	0.034
	HQ	0.10	0.26	0.05	0.07	0.09
**Market Samples**	Consumption (g/day)	27	50	25	40	50
	Patulin (μg/kg)	95.7	145.7	50.6	45.5	44.8
	Dietary Intake (μg/kg/day)	0.039	0.11	0.019	0.027	0.033
	HQ	0.10	0.28	0.05	0.07	0.08

Mean weight = 66 ± 1.5 kg.

## 4 Discussion

### 4.1 Patulin levels in fruits

The levels of patulin in fruits in the current study were lower as contrasted to our prior study [25], which verified that 57.4 % of samples of fruits have levels (≥LOD), varying from 0.04 to 1100 μg/kg. The maximum average value of patulin was observed in red globe grapes 921.1 ± 22.4 μg/kg. In another study [29], the patulin levels were documented in different varieties of apples. The results have revealed that the maximum average value of patulin was found in golden apples and juices, i.e., 310.8 ± 21.5 μg/kg and 290.8 ± 14.6 μg/kg, respectively (higher levels compared to the results of current findings). The level of patulin was documented in 2970 citrus fruit samples, and 31.7 % of samples have values of patulin of 0.12–320 μg/kg, and 22.1 % of samples have levels of patulin greater than the maximum limit of European Union (50 μg/kg) [26].

Other studies have documented that 12–52 % of juices were contaminated with patulin [30,31]. Patulin levels were found in 12 % of 177 apple juice samples, and 1 % are above the maximum 50 μg/kg [30]. Furthermore, it was evident from research findings that organic apple juice contained more patulin levels than conventional ones. Harris et al. [31] have observed that 23 % of apple juice samples comprised patulin levels ranging from 8.8 to 2700 μg/L, and 11.3 % samples have concentrations greater than 50 μg/kg. Patulin levels were documented in 18 % of juice samples, of which 30 were apple juice and 30 were mixed with levels higher than the recommended limit [32]. In China, patulin concentrations were discovered in fruit products (fruit juice, dried fruits, and jams), extending from 10 to 276.9 μg/kg [4]. Similarly, from China, the findings documented that patulin's maximum value was 94.7 μg/kg in apple juice samples, with 20 % of samples having levels elevated than the advocated limit [33].

The detection of mycotoxins within the supply chain system is a valuable tool for managing and mitigating the presence of mycotoxins in the food chain system. According to Heperkan et al. [34], an elevated concentration of patulin was seen in fruits directly obtained from the orchards owned by the growers. The growth of fungus during storage is primarily influenced by factors such as the nutritional makeup of food, temperature and moisture conditions, and the presence of stored insects [35]. The quantities of moisture and temperature present in food commodities primarily influence the development of mycotoxins. According to Channaiah [36], the optimal conditions for mold growth typically involve temperatures ranging from 10 to 40.5 °C, relative humidity levels exceeding 70 %, and a pH range between 4 and 8. In addition, relative humidity is a significant component that influences moisture levels in storage conditions. The lack of control over moisture and temperature conditions in transit may potentially contribute to elevated amounts of patulin in samples obtained during transportation.

### 4.2 Dietary intake of patulin

Our previous study documented lower dietary intake levels of patulin, i.e., 0.0049, 0.0016, and 0.0014 μg/kg/day in fruits, juices, and smoothies, with HQ levels of 1.22, 0.40, and 0.35, respectively. In another study from Qatar, the dietary intake levels ranged between 7.2 and 74.5 ng/kg bw/day for apple juice samples and 1.3–36 ng/kg bw/day for apple samples. The HQ levels were determined in apple-based baby foods, with values ranging from 0.244 to 0.27 [13]. The dietary intake in children, adults, and adolescents was determined in juices and documented the dietary intake of 25.9–50.6 ng/kg bw/day for children and 2.8–5.5 ng/kg bw/day for adults [23]. Assuncao et al. [37] documented the patulin exposure assessment in cereal-based foods and found a dietary intake of 3.59–22.93 ng/kg bw/day.

## 5 Conclusion

Current research findings show comparatively high levels of patulin in selected fruits but lower than those in our previous studies. The highest mean patulin levels were 77.10 ± 44.4 μg/kg in fruits collected from the market. Samples 56, 41, and 38 of fruits have patulin levels greater than 50 μg/kg from samples collected during transportation, market samples, and farm samples, respectively. PAT's highest mean dietary intake was 0.11 μg/kg bw/day in apple samples, and the highest mean hazard quotient (HQ) value of 0.28. The results emphasized the importance of regular and updated surveys by food regulation agencies and disseminating related information to traders, exporters, farmers, and consumers. In the current work, an effort was made to investigate the traceability of patulin in juice samples during the processing step from field to table, which was quite different from other research works on the same topic. However, there are some limitations in terms of the analytical facilities that are available. It would be much better if LC/MS or UHPLC were used to detect multi-mycotoxins in fruits and juices.

## Data availability

Data included in article/supp. Material/referenced in the article.

## Ethics approval

Not applicable.

Consent to participate.

All participants have given their consent to participate in this study.

## CRediT authorship contribution statement

**Shahzad Z. Iqbal:** Project administration, Conceptualization. **Muhammad Waseem:** Methodology, Investigation. **Ahmad Faizal Abdull Razis:** Resources, Data curation. **Ijaz A. Bhatti:** Writing – review & editing, Conceptualization. **Amin Mousavi Khaneghah:** Visualization, Software. **Osama A. Mohammed:** Writing – review & editing, Resources, Funding acquisition. **Srimathi P. Lakshminarayanan:** Software, Formal analysis. **Munawar Iqbal:** Writing – review & editing, Visualization.

## Declaration of competing interest

The authors declare that they have no known competing financial interests or personal relationships that could have appeared to influence the work reported in this paper.
