# Supplementary material for: Mycotoxin patulin contamination in various fruits and estimating its dietary impact on the consumers: From orchard to table
Source: Heliyon. 2024 Apr 28;10(10):e30252. doi: 10.1016/j.heliyon.2024.e30252 (PMC11109727; doi:10.1016/j.heliyon.2024.e30252)
Supplement: Multimedia component 1 [file mmc1.docx]

**Food Frequency Questionnaire**

**Title:** Mycotoxin Patulin Contamination in Various Fruits and Estimating Its Dietary Impact on the Consumers: From Orchard to Table

| **Food and Amount** | **Average Use Last Year** | | | | | | |
| --- | --- | --- | --- | --- | --- | --- | --- |
| **Sweet potato**  **Ready-to eat** | **Never or Less than 1 grams** | **1-50 mL** | **50-100 mL** | **100-200 mL** | **200- 400 mL** | **400- 500 mL** | **Once a day**  **250 mL** |
| **Apple juice** |  |  |  |  |  |  |  |
| **Fruit juices** |  |  |  |  |  |  |  |
|  |  |  |  |  |  |  |  |
|  |  |  |  |  |  |  |  |
|  |  |  |  |  |  |  |  |

**Questions**

2: Are their any other diet which you eat during day…………….. if yes please mention all those which grams.

---------------------------------------------------------------------------------------------------------------------

3. How much water did you drink or other liquids such as tea, coffee, or milk ?

-------------------------------------------------------------------------------------------------------

4: What kind of fat mostly did you include in your diet?

------------------------------------------------------------------------------------------------------------------

5. Have you taken any vitamins, supplements etc? if yes please mention those and include their amounttoo??

**Participant ID**: **Participant age**: **Participant weight**:

**Gender:**

Participant consent: Yes / No
